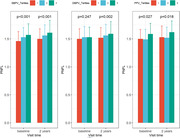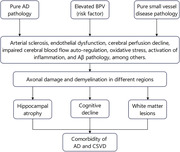# Association of long‐term blood pressure variability with plasma neurofilament light underlies the comorbidity of Alzheimer's Disease and cerebral small vessel disease

**DOI:** 10.1002/alz.085204

**Published:** 2025-01-03

**Authors:** Qin Li, Xiaofeng Li

**Affiliations:** ^1^ The Second Affiliated Hospital of Chongqing Medical University, Chongqing China; ^2^ The Second Affiliated Hospital of Chongqing Medical University, Chongqing, Chong Qing China

## Abstract

**Background:**

Alzheimer’s disease (AD) frequently coexists with cerebral small vessel disease (CSVD) is common in the aging population, yet the underlying mechanisms are not yet fully understood. Both long‐term blood pressure variability (BPV) and plasma neurofilament light (PNFL) were identified as potential biomarkers for AD and CSVD. This study aims to understand the mechanisms of comorbidity between AD and CSVD by investigating the associations among BPV, PNFL, and comorbidity. The severity of CSVD was visualized as white matter hyperintensities (WMH).

**Method:**

Data was collected from the Alzheimer’s Disease Neuroimaging Initiative database. Participants with cognitively normal and mild cognitive impairment were included in the data analyses as clinical dementia rating (CDR) = 0 and 0.5. Aβ status was determined by cerebrospinal fluid Aβ levels and the WMH burden was defined by adjusting the WMH volume for intracranial volume. The differences in BPV and PNFL levels among participants with different Aβ status and varying WMH burdens were compared. Causal mediation analyses were conducted to explore whether the relationships of BPV with brain structural changes and cognition were mediated by PNFL.

**Result:**

Aβ positive participants with high WMH burden had higher BPV and PNFL compared to Aβ negative individuals with low WMH burden. Systolic BPV (estimate [est] = 0.022, p = 0.004) was associated with WMH volume, and diastolic BPV (est = ‐0.053, p = 0.002) was associated with hippocampal volume. Both systolic BPV and diastolic BPV were associated with memory and executive function. Moreover, systolic (est = 0.013, p<0.001) and diastolic BPV (est = 0.012, p < 0.001) were associated with PNFL independent with average blood pressure. Mediation analyses revealed that PNFL mediated the association between systolic BPV and both WMH volume and executive function. PNFL also mediated the relationship between diastolic BPV and both hippocampal volume and memory function.

**Conclusion:**

Elevated BPV was linked with axonal damage, hippocampal atrophy, WMH progression, and cognitive decline. This finding suggests that the same risk factor may result in pathological changes in different brain regions and cognitive decline, which in turn may be associated with the comorbidity of AD and CSVD.